# Structural and functional consequences of removing the N-terminal domain from the magnesium chelatase ChlH subunit of *Thermosynechococcus elongatus*

**DOI:** 10.1042/BJ20140463

**Published:** 2014-12-05

**Authors:** Nathan B. P. Adams, Christopher J. Marklew, Pu Qian, Amanda A. Brindley, Paul A. Davison, Per A. Bullough, C. Neil Hunter

**Affiliations:** *Department of Molecular Biology and Biotechnology, University of Sheffield, Sheffield S10 2TN, U.K.

**Keywords:** chlorophyll, chlorophyll biosynthesis, electron microscopy, magnesium chelatase, *Synechocystis* sp. PCC6803, *Thermosynechococcus elongatus*, Chl, chlorophyll, β-DDM, *n*-dodecyl-β-D-maltopyranoside, MgCH, magnesium chelatase, Proto, protoporphyrin IX, MgProto, magnesium protoporphyrin, D_IX_, deuteroporphyrin, MgD_IX_, magnesium deuteroporphyrin, MRA, multi-reference alignment

## Abstract

Magnesium chelatase (MgCH) initiates chlorophyll biosynthesis by catalysing the ATP-dependent insertion of Mg^2+^ into protoporphyrin. This large enzyme complex comprises ChlH, I and D subunits, with I and D involved in ATP hydrolysis, and H the protein that handles the substrate and product. The 148 kDa ChlH subunit has a globular N-terminal domain attached by a narrow linker to a hollow cage-like structure. Following deletion of this ~18 kDa domain from the *Thermosynechoccus elongatus* ChlH, we used single particle reconstruction to show that the apo- and porphyrin-bound forms of the mutant subunit consist of a hollow globular protein with three connected lobes; superposition of the mutant and native ChlH structures shows that, despite the clear absence of the N-terminal ‘head’ region, the rest of the protein appears to be correctly folded. Analyses of dissociation constants shows that the ΔN159ChlH mutant retains the ability to bind protoporphyrin and the Gun4 enhancer protein, although the addition of I and D subunits yields an extremely impaired active enzyme complex. Addition of the Gun4 enhancer protein, which stimulates MgCH activity significantly especially at low Mg^2+^ concentrations, partially reactivates the ΔN159ChlH–I–D mutant enzyme complex, suggesting that the binding site or sites for Gun4 on H do not wholly depend on the N-terminal domain.

## Abstract 2

The N-terminal domain of the 148 kDa ChlH is essential for normal activity of the ChlH–I–D magnesium chelatase complex. Deleting this 18 kDa domain retains the hollow cage-like structure and porphyrin binding. Chelatase activity is partially restored by the Gun4 protein.

## INTRODUCTION

Chlorophylls (Chls) are the essential cofactors for photosynthesis, and their biosynthesis involves a series of enzyme-driven reactions, the first committed step of which is catalysed by magnesium chelatase (E.C. 6.6.1.1; MgCH). This three-subunit enzyme (H ~150 kDa; D ~70 kDa; I ~40 kDa) is situated at the branch-point between Chl and haem biosynthesis, and it catalyses the ATP-dependent insertion of a Mg^2+^ ion into the protoporphyrin IX (Proto) macrocycle [1,2]. H is the porphyrin-binding subunit [3,4]. The I subunit (BchI in purple phototrophic bacteria, ChlI in cyanobacteria and plants) is an active ATPase [5,6] that associates with the D subunit [5,7]. The D subunit is not an ATPase, but the nucleotide binding site is required for allosteric regulation of MgCH [8]. I and D are members of the AAA^+^ super-family, and cryo-EM of the BchI–D–MgATP complex showed that BchI and BchD form a double I_6_D_6_ ring [9]. The cyanobacterial MgCH from *Synechocystis* sp. PCC6803 has been analysed by steady-state [5,10–13] and transient [14] kinetic approaches, as has the thermostable enzyme from *Thermosynechoccus elongatus* [15]. The catalytic cycle is thought to involve the association of H-Proto and IDMgATP, forming a short-lived complex that couples the hydrolysis of ATP to the formation of magnesium protoporphyrin (MgProto).

The 2.1 Å (1 Å=0.1 nm) structure of the BchI subunit from the purple phototroph *Rhodobacter capsulatus* [16] has been used to interpret models of the BchI_6_D_6_ complex calculated from single-particle cryo-EM reconstructions [9]. These models, obtained using subunits incubated with either ADP or ATP, show that the catalytic cycle of MgCH involves conformational changes in the ID units. Rather less is known about the H subunit, although a low-resolution structural model of BchH from *Rba. capsulatus* has been obtained through 3D reconstruction of negatively stained single particles [17], and a similar approach, augmented by SAXS, was used to determine a low resolution structure of the cyanobacterial ChlH subunit [18]. In contrast with the more open BchH structure, ChlH forms a more enclosed cage-like assembly, connected to an N-terminal ‘head’ region adjacent to a 5 nm-diameter opening in the structure. It was suggested that the more enclosed ChlH structure affords some protection for the labile MgProto product, given the potentially damaging combination of light and oxygen within cells of oxygenic phototrophs such as cyanobacteria and plants [18]. A more complex structure for the ChlH with respect to BchH could also be a consequence of interactions with the Gun4 protein [19,20], which stimulates Mg-chelatase at low magnesium concentrations [21]. The structure of the cyanobacterial Gun4 protein has been determined by X-ray crystallography [21,22], but there is currently no structural detail available for the ChlH–Gun4 complex.

The N-terminal ‘head’ domain of ChlH of *T. elongatus* was identified by binding NTA–Nanogold to the N-terminal His_6_ tag [18]. This domain, estimated with the ‘neck’ to have a molecular mass of ~17.6 kDa, is of interest given its location adjacent to the entrance to the lumen within ChlH and its connection to the main body of ChlH through a narrow linker region between Gly^127^ and Phe^156^. We speculated that the head domain, together with a flexible linker, could be involved in gating access to the cavity within ChlH [18]. In the present study, we removed 159 residues from the N-terminus of ChlH from *T. elongatus* to find out whether this head domain is indeed at the N-terminus, and to determine whether it represents an autonomous region of ChlH, in the sense that the rest of the ChlH folds correctly in its absence. The low-resolution structures of the apo- and porphyrin-bound forms of the ΔN159ChlH subunit show that the N-terminal domain has been removed cleanly from the rest of the protein, and functional assays show that the remaining 130 kDa of this N-terminally truncated subunit still binds the porphyrin substrate, the product of the MgCH reaction, and the Gun4 enhancer protein. Only 10% of chelatase activity remains following removal of the head domain, but this value increases to 24% in the presence of saturating concentrations of Gun4. We discuss the possible functional roles of the head domain of ChlH.

## EXPERIMENTAL

### Construction of *T. elongatus* ΔN159chlH

The truncated *chlH* gene was engineered by introducing a new NdeI site into the DNA at Met^159^ using the following primers: M159TOP, 5′-TGCCGGCTTCCAAGAT-CATATGCTCAAACTCC-3′ and M159BOT, 5′-GGAGTTT-GAGCATATGATCTTGGAAGCCGGCA-3′. QuikChange® PCR was carried out according to the manufacturer's protocol (Stratagene), using the *T. elongatus* cloned pET9aHis_6_ChlH as the template. The PCR product was digested with NdeI, gel purified and ligated with T4 DNA ligase (Promega). The resultant pET9aHis_6_ΔN159*chlH* construct was verified by DNA sequencing (GATC) prior to overexpression.

### Purification of recombinant ΔN159ChlH from *T. elongatus*

The plasmid containing the gene encoding *T. elongatus* ΔN159ChlH was transformed into *Escherichia coli* and the cells were induced for protein production as described by Qian et al. [18], then harvested after 4 h expression. Initial purification revealed that most of the protein was in a highly aggregated state. Monomeric protein was produced by adding *n*-dodecyl-β-D-maltopyranoside (β-DDM) to the supernatant (and all buffers) to a final concentration of 0.05% and gently incubating for 1 min in a sonicating water bath prior to purification. The purification was performed as described in Qian et al. [18], using immobilized metal affinity chromatography, then ion-exchange chromatography on Q-Sepharose and finally HPLC gel filtration.

### Purification of recombinant *T. elongatus* ΔN159ChlH for MgCH assays

The gene encoding ΔN159ChlH was overexpressed in *E. coli* strain Rosetta 2 *pLysS* (Novagen). Cultures in LB (500 ml) were grown to a *D*_600_ of 0.6 at 37°C then transferred to 20°C for 2 h with 0.4 mM IPTG. The purification was performed according to Qian et al. [18], using immobilized metal affinity chromatography, ion-exchange chromatography on Resource Q (GE Healthcare), and finally gel filtration on a Superdex 200 (GE Healthcare). Binding of deuteroporphyrin (D_IX_) to ΔN159ChlH was performed using the method in Qian et al. [18].

### Recombinant *T. elongatus* Gun4 protein

The *gun4* gene from *T. elongatus* was cloned into pET14b by PCR with primers TEG4NdeI, 5′-TCCATATGATGGT-CACCACAGAACCAGCCTTAGC-3′ and TEG4BamHI, 5′-TCGGATCCCTAGGCGGTCCAGGCAGGATGATTAAGG-3′ using *T. elongatus* pGEX-4T-1Gun4 as the template. The PCR product was digested with NdeI and BamHI and ligated into pET14b cut with the same restriction enzymes. The resultant pET14bTEGun4 construct was verified by DNA sequencing (GATC) prior to overexpression. pET14bTEGun4 was transformed into *E. coli* strain Rosetta 2 *pLysS* for overexpression of the Gun4 proteins. Cultures in LB (500 ml) were grown to a *D*_600_ of 1 at 37°C then transferred to 20°C overnight with 0.4 mM IPTG. The proteins were purified by metal affinity chromatography and buffer exchanged into 50 mM Tricine (pH 7.9), 0.2 M NaCl, 0.3 M glycerol prior to MgCH assays.

### Electron microscopy

Purified proteins were adsorbed on to freshly glow-discharged carbon-coated copper grids and negatively stained as described in [18]. In order to obtain a suitable protein particle density on EM grids, the purified ΔN159ChlH protein was diluted using HPLC running buffer (50 mM Tricine/NaOH (pH 7.9), 0.3 M NaCl, 0.3 M glycerol, 1 mM DTT, 0.05% β-DDM). Carbon-coated (~159 Å thickness) 400 mesh copper EM grids (Agar Scientific) were glow-discharged for 30 s. The diluted protein solution (5 μl) was applied to a grid and left for 30 s before gentle blotting with filter paper. The grid then was washed twice with water and stained with 0.75% uranyl formate solution for 20 s. Excess stain solution was removed by blotting then drying in air. Data were collected using a Philips CM-100 microscope fitted with a 1K×1K Gatan Multiscan 794 CCD camera with magnification set at ×61000, which corresponds to 3.93 Å per pixel at the specimen level; the underfocus value was adjusted to ∼1.0 μm. Tilt pair images were recorded by turning the sample holder around the tilt axis by 0° and 10°.

### Data processing

Single particles of ΔN159ChlH were picked semi-automatically from the electron micrographs using EMAN2 [23], with a square box of 56 pixels×56 pixels corresponding to 22 nm×22 nm. 20236 combined particles were then treated subsequently using the IMAGIC-5 software package [24]. All particles were band-pass filtered to suppress low spatial frequencies, initially according to the suggested values (low frequency cut-off 0.04; high frequency cut-off 0.8) in the IMAGIC-5 operation manual (http://www.imagescience.de/manuls/smii.pdf). The filtered particles were masked, normalized and centred for reference-free 2D classification; a set of 25 characteristic 2D classes was used for multi-reference alignment (MRA) and multivariate statistical analysis [25,26]. A few iterations were performed until a stable 2D classification was obtained. A total of 600 2D classes were selected for Euler angle assignment and subsequent 3D reconstruction. An initial 3D model was produced using the e2initialmodel.py programme in the EMAN2 software package. This 3D model was used to calculate the Euler angles of the averaged 2D classes; a new model was produced from the data set and re-projected so new Euler angles could be re-assigned. This iteration was continued until a stable 3D model was obtained.

Similar treatments were applied for the 3D reconstruction of porphyrin-bound ΔN159ChlH. Here, 18659 particles were picked from 220 electron micrographs.

### Binding of substrate and product to ΔN159ChlH

Tryptophan quenching assays and calculations of *K*_d_ values for the D_IX_ and magnesium deuteroporphyrin (MgD_IX_) ligands were performed as described previously [3].

### Labelling proteins

Gun4 was reduced in 10 mM DTT for 10 min at room temperature before buffer exchange into 20 mM NaH_2_PO_4_ and 150 mM NaCl, pH 7.4, using a Zeba Spin column (Thermo Scientific) and then labelled with a 10-fold excess of Alexa Fluor 532 C_5_ maleimide (Life Technologies), dissolved in dry DMF, for 2 h at room temperature while being protected from the light. The reaction was quenched by the addition of 10 mM DTT for 15 min.

Wild-type (WT) ChlH and ΔN159ChlH were desalted into PBS titrated to pH 8.3 with 0.2 M pH 9 sodium bicarbonate, using a Zeba Spin column. Protein was labelled with a 3-fold excess of Atto 488 NHS ester dissolved in dry DMF for 1 h at room temperature while being protected from light. The reaction was quenched with the addition of 10 mM Tris/HCl (pH 7.4) for 15 min.

Proteins were separated from excess dye by using a PD-10 desalting column (GE Healthcare) equilibrated in 50 mM Tricine/NaOH, 200 mM NaCl and 0.3 M glycerol.

### Monitoring protein association via FRET

Assays contained 0.1 μM WT ChlH or ΔN159ChlH labelled with Atto 488 in 50 mM Tricine/NaOH, 0.3 M glycerol and 200 mM NaCl, pH 7.7, at 45°C. Fluorescence emission spectra were recorded by exciting at 450±2.5 nm. Fluorescence quenching of the emission signal for ChlH was monitored at 524 nm, corrected by subtracting the emission of Gun4 in the absence of ChlH and reported as a function of [Gun4]. The quenching of the signal at 524 nm was deemed to be due to quenching by FRET to Gun4 labelled with Alexa Fluor 532. Non-linear regression was performed using Igor Pro 6.32 (Wavemetrics).

### MgCH enzyme assays

ChlI, ChlD and ChlH were purified as described previously [15]. Assays were performed in 50 mM Mops/KOH, 0.3 M glycerol, 15 mM MgCl_2_, 5 mM ATP, 8 μM D_IX_, *I*=0.1 (KCl), at 45°C pH 7.7. Assays were initiated by the addition of enzyme, with 0.1 μM ChlD, 0.2 μM ChlI and 0.4 μM ChlH WT or ΔN159ChlH, and various concentrations of Gun4.

Steady-state assays of MgCH were performed using a BMG LabTech Omega FluoStar microplate reader, with excitation through a 420±5 nm filter. Fluorescence emission, using a 580±5 nm filter, detected the production of product MgD_IX_ over a period of 1–2 h. Steady-state rates (*v*_ss_) were calculated using the plate reader software (MARS Data analysis suite version 2.41) taking the steepest gradient over a 10 min time interval. Calibration was performed using known concentrations of MgD_IX_ in the presence of standard concentrations of subunits (ChlI, D and H) and the data were fitted to a second order polynomial. Non-linear regression was performed using Igor Pro. The concentration of porphyrins was determined by visible spectroscopy in 0.1 M HCl with the molar absorption coefficient of 433000 M^−1^·cm^−1^ at 399 nm. The concentration of ATP in water was determined using the molar absorption coef-ficient of 15400 M^−1^·cm^−1^ at 260 nm. Concentrations of proteins were determined according to their absorbance at 280 nm with the following molar absorption coefficients: ChlI, 15640 M^−1^·cm^−1^; ChlD 30230 M^−1^·cm^−1^; ChlH 154600 M^−1^·cm^−1^; ΔN159ChlH 148080 M^−1^·cm^−1^; Gun4 67380 M^−1^·cm^−1^.

In eqn (1), *F*_obs_ is the observed fluorescence, *F*_0_ is the initial fluorescence, *F*_min_ is the minimum amplitude of fluorescence quenching and *K*_d_ is the apparent dissociation constant.

In eqn (2), *v*_ss_ is the observed steady-state rate, *v*_0_ is the steady-state chelatase rate in the absence of Gun4, *v*_lim_ is the limiting steady-state rate reached at saturating Gun4, *n*_d_ is the concentration of binding sites for Gun4, i.e. ([ChlH]×(number of sites/ChlH), and *K*_app_ is the apparent dissociation constant for the active ChlH–Gun4 complex.

(1) $$F_{\mathrm{obs}}=F_{0}-F_{\min}\frac{\left[\mathrm{Gun}4\right]+\left[\mathrm{ChlH}\right]+K_{d}-\sqrt{{\left(\left[\mathrm{Gun}4\right]+\left[\mathrm{ChlH}\right]+K_{d}\right)}^{2}-4\left[\mathrm{ChlH}\right]\left[\mathrm{Gun}4\right]}}{2\left[\mathrm{ChlH}\right]}$$

(2) $$V_{\mathrm{ss}}=V_{0}+V_{\lim}\frac{\left[\mathrm{Gun}4\right]+n_{d}+K_{\mathrm{app}}-\sqrt{{\left(\left[\mathrm{Gun}4\right]+n_{d}+K_{\mathrm{app}}\right)}^{2}-4n_{d}\left[\mathrm{Gun}4\right]}}{2n_{d}}$$

## RESULTS

### Choice of the ΔN159ChlH truncation, and purification of the recombinant *T. elongatus* ΔN159ChlH protein

**Figure 1:**
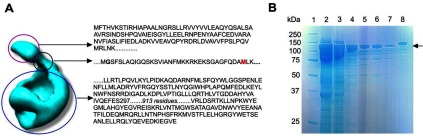
The proposed head, linker and main regions of ChlH; purification of the *T. elongatus* ΔN159ChlH mutant lacking the head and linker regions (**A**) 3D structural model of the apo-ChlH MgCH subunit from *T. elongatus*, calculated at 30 Å resolution [18]; the indigo circle delineates the N-terminal head domain, connected to the rest of ChlH (blue circle) by a narrow linker (black circle). Amino acids predicted to comprise the three parts of ChlH are shown to the right of the model. The glycine residue highlighted in bold after the first methionine residue in the linker sequence is conserved in all known ChlH/BchH proteins. The methionine residue in red in the linker sequence was chosen as the initiation codon for the ΔN159ChlH. (**B**) SDS/PAGE of purified recombinant ΔN159ChlH. 1, Protein markers; 2, supernatant fraction from cell extract; 3, flow-through from binding supernatant to Ni^2+^ affinity column; 4, pooled eluted fractions containing ΔN159ChlH; 5, pooled eluted fractions containing ΔN159ChlH following anion-exchange chromatography; 6, ΔN159ChlH fractions from size-exclusion chromatography; 7, ΔN159ChlH fractions following HPLC size-exclusion chromatography; 8, WT ChlH standard. The arrow indicates the ΔN159ChlH band, estimated as 135 kDa.

The N-terminal domain had been identified previously by attaching a 5 nm diameter NTA–Nanogold particle to the N-terminal His_6_ tag, and imaging the labelled molecules using EM [18]. The molecular mass of the N-terminal ‘head’ domain of ChlH, connected to the rest of ChlH by a narrow linker, was estimated to lie within the range 14.1–17.3 kDa [18]. Thus, the sequence MG^127^SFSLAQIG^135^QSKSVIANFMKKRKEKSG^153^AG^155^FQDAMLK (Figure 1A) is likely to contain the linker region. It was noted in [18] that Gly^127^ is conserved in all MgCH H subunits, and that between three and five glycine residues are generally found in this region of ChlH proteins, consistent with a degree of flexibility in the linker and a possible gating role for the ‘head’ region. Truncation using Met^126^ as the start codon conserved the linker, but this construct did not yield any recombinant protein; using Met^160^ instead for the truncated ChlH mutant designated ΔN159ChlH did yield protein, found in both the soluble and insoluble fractions of disrupted *E. coli* cells. Expression trials (results not shown) were performed to limit the aggregation of truncated protein and, following purification using immobilized metal affinity chromatography then ion-exchange chromatography, we monitored the aggregation state of the ΔN159ChlH protein using HLPC gel filtration. It was found that exchanging ΔN159ChlH into buffer containing the detergent β-DDM maintained nearly all the truncated ChlH in a monomeric state. It is likely that removal of the N-terminal domain and the linker exposes a hydrophobic patch, which can be shielded by detergent molecules. Subsequently, therefore, 0.05% β-DDM was added to the supernatant and to all buffers used for ΔN159ChlH ChlH.

Recombinant His_6_-tagged ΔN159ChlH from *T. elongatus* was purified using a three-step procedure involving immobilized metal affinity chromatography, ion-exchange chromatography, and gel filtration. For protein analysed by single particle analysis (see next section), an additional HPLC gel filtration step was included. The SDS/PAGE analysis of ΔN159ChlH, monitored through each of the purification steps, shows a high level of purity after gel filtration (Figure 1B). As expected, removal of the N-terminal domain reduces the molecular mass of ΔN159ChlH, in comparison with the ChlH standard, which has a predicted molecular mass of 148 kDa.

### Single particle analysis of the ΔN159ChlH and porphyrin-bound ΔN159ChlH proteins

**Figure 2:**
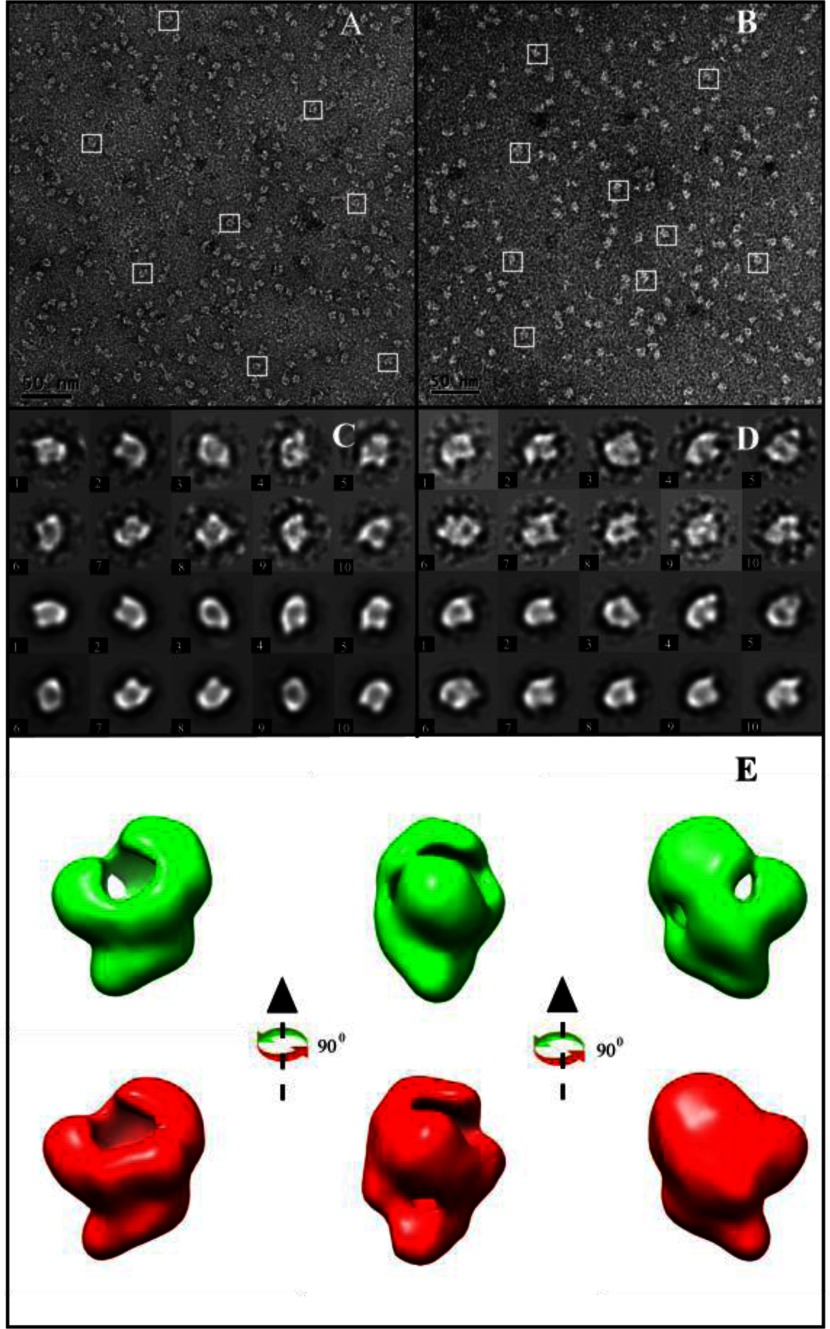
EM raw data and classifications with 3D reconstruction of *T. elongatus* ΔN159ChlH (**A**) Electron microscopy of purified ΔN159ChlH with the white boxes indicating single proteins. Scale bar=50 nm. (**B**) Electron micrograph of purified D_IX_-bound ΔN159ChlH. (**C**) Gallery of selected 2D averaged classes (top two rows) and their corresponding reprojections (bottom two rows) from 3D models of ΔN159ChlH. The box size is 22 nm×22 nm. (**D**) Gallery from porphyrin-bound ΔN159ChlH. (**E**) 3D reconstruction of ΔN159ChlH without (green) and with (red) bound porphyrin, rotated through 90°. The 3D models were generated using Chimera [33].

Figures 2(A) and 2(B) show typical raw data for the clearly monomeric ΔN159ChlH and D_IX_-bound ΔN159ChlH proteins. Approximately 70 particles could be identified within a single 1K × 1K micrograph, and in total 20236 and 18659 particles were picked for further analysis (see Experimental section). The top two rows of Figures 2C and 2D show examples of averaged 2D classes from ΔN159ChlH and porphyrin-bound ΔN159ChlH proteins, and for comparison the bottom two rows display the reprojections from the stable 3D models, which are consistent with the corresponding 2D classes. Supplementary Figure S1 shows the distribution of Euler angles used for our image analyses of the ΔN159ChlH and porphyrin-bound ΔN159ChlH protein samples. A total of 600 2D averaged classes was selected for ΔN159ChlH and porphyrin-bound ΔN159ChlH, and compared with WT ChlH (apo-ChlH, 75 classes; substrate-bound ChlH, 92 classes; [18]); the absence of the ‘head’ region presumably allows a better 3D distribution of the protein orientations.

The reconstructed 3D models of the ΔN159ChlH and D_IX_-bound ΔN159ChlH protein are displayed in Figure 2E, twice rotated about the *z*-axis by 90°. The threshold values for the volume of these models were adjusted to their predicted molecular mass of 130 kDa. The ΔN159ChlH and porphyrin-bound ΔN159ChlH structures, calculated to 23 and 27 Å, respectively (Supplementary Figure S2), both show a hollow globular protein with three connected lobes enclosing a large open lumen. There are no significant differences between these two structures at these low resolutions.

**Figure 3:**
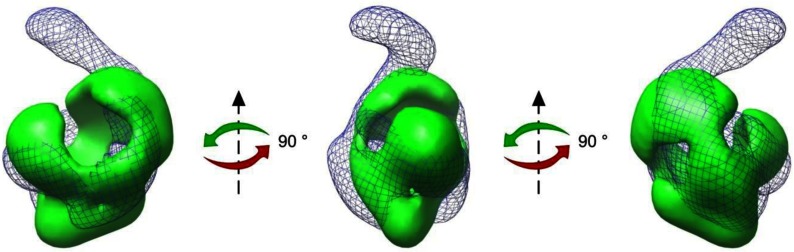
Superposition of the 3D structures of *T. elongatus* ΔN159ChlH and WT ChlH Superposition of the WT ChlH structure [18] (blue wire mesh model) on to the 3D model for the ΔN159ChlH (green solid–without porphyrin) rotated through 90° successively about the *z*-axis.

Figure 3 shows the superposition of the ΔN159ChlH mutant on to the previously published *T. elongatus* ChlH model [18]. The choice of handedness and best fit was judged by eye, but we emphasize that the absolute hand of the two structures is unknown. The two models are not identical in their globular regions, which may be the result of a slight alteration in folding or it may be attributed to the experimental error associated with negative staining. However, the superposition shows that the main globular regions of the truncated and WT ChlH proteins are of a similar size and that the gaps and apertures in the structure are approximately in the same positions. The most striking and significant difference is the clear absence of the WT ‘head’ region from the ΔN159ChlH mutant.

### Porphyrin binding studies and the effect of Gun4 on ΔN159ChlH in MgCH assays

**Figure 4:**
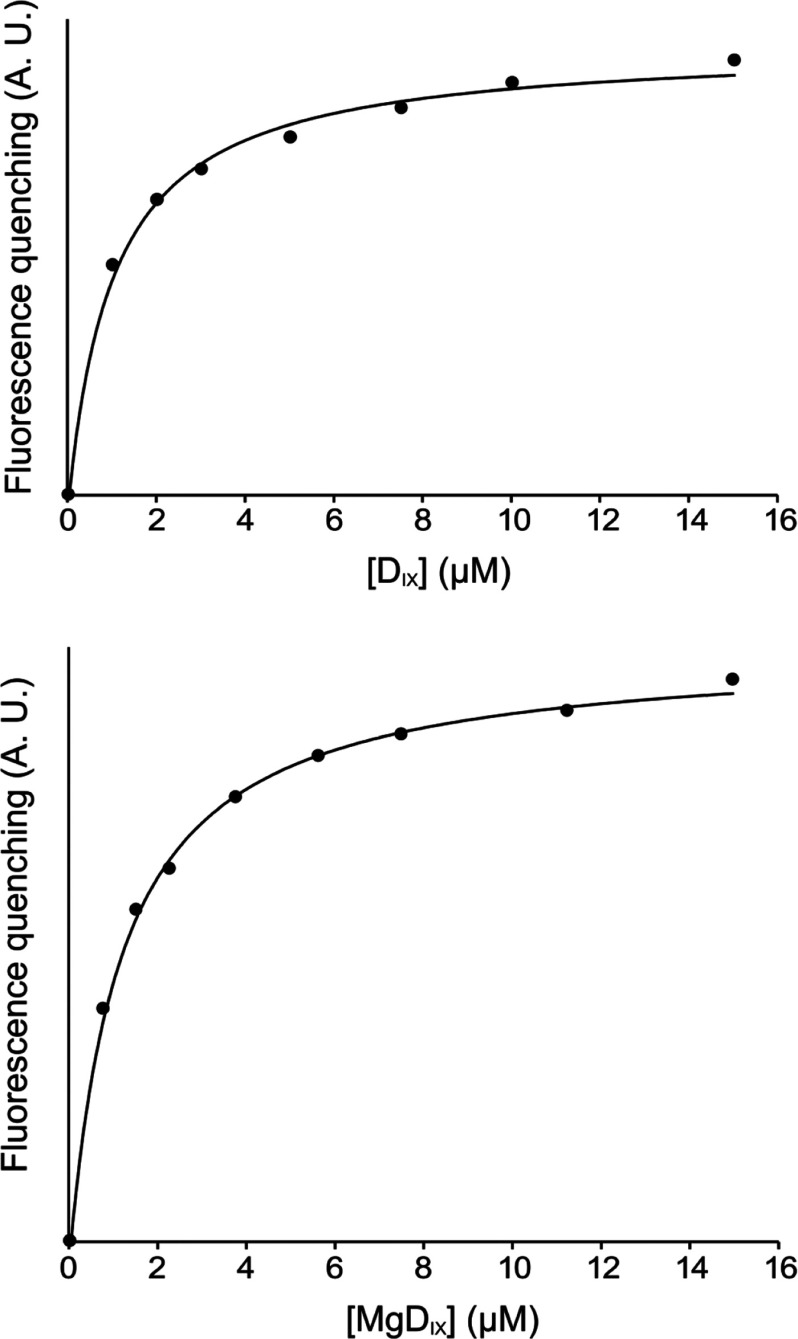
Substrate and product binding curves for *T. elongatus* ΔN159ChlH Quenching of ΔN159ChlH protein fluorescence of; top, deuteroporphyrin IX; bottom, MgD_IX_. 0.1 μM of purified ΔN159ChlH was incubated with 0–15 μM substrate or product and incubated at 34°C. Using the excitation wavelength of 295 nm, fluorescence emission scans were recorded at 34°C. A.U., arbitrary units. Both curves show single-site binding. The *K*_d_ values are 1.06±0.09 and 1.64±0.08 μM, respectively.

Given the apparent preservation of the rest of the ChlH structure, following removal of the N-terminal ‘head’ domain, it was of interest to see whether the porphyrin binding and catalytic functions had been retained in the ΔN159ChlH mutant. The fluorescence of ChlH tryptophan residues is quenched upon binding of porphyrins, enabling dissociation constants between protein, substrate and product to be determined [3]. Figure 4 displays plots of integrated fluorescence as a function of tetrapyrrole concentration, with the data fitted to a single substrate binding model [3]. The calculated *K*_d_ values of ΔN159ChlH for D_IX_ substrate and MgD_IX_ product were 1.06±0.09 and 1.64±0.08 μM, respectively; these values are consistent within the dissociation constants for WT *T. elongatus* ChlH of 1.48±0.3 μM for D_IX_ [18] and 2.12±0.2 μM for MgD_IX_ (see Supplementary Figure S3).

**Figure 5:**
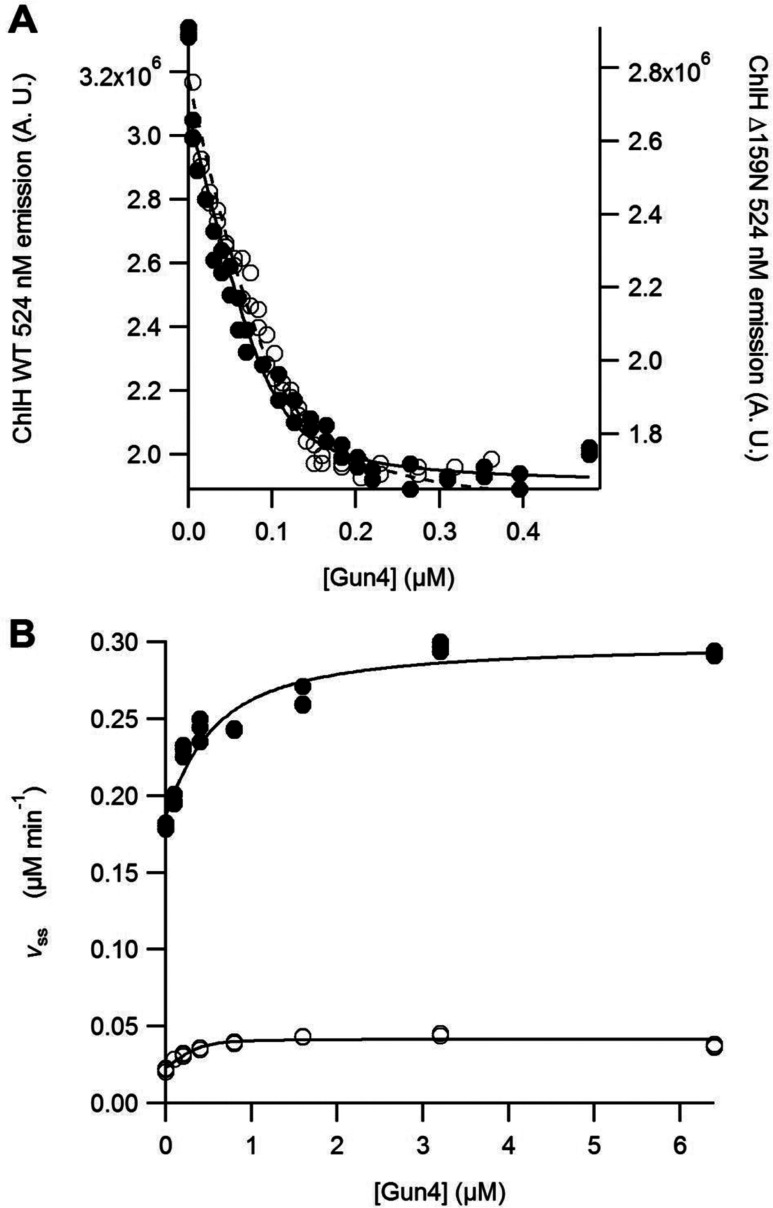
Gun4 is able to associate with and stimulate activity of ΔN159ChlH (**A**) FRET association experiment between Gun4 and WT ChlH (●) or ΔN159ChlH (○). Assays contained 0.1 μM ChlH, in 50 mM Tricine/NaOH, 0.3 M glycerol and 200 mM NaCl, pH 7.9, at 45°C. ChlH was labelled with Atto 488 and Gun4 labelled with Alexa Fluor 532. The quenching of emission of Atto 488 was monitored at 524 nm. A.U.–arbitrary units. The curves can be described by eqn (1) with the concentration term, [ChlH], held at 0.1 μM with characterizing parameters: WT ChlH *K*_d_=0.009±0.003 μM and Δ159ChlH *K*_d_=0.016±0.004 μM. (**B**) Assembly titrations of MgCH between Gun4. Assays contained 0.1 μM ChlD, 0.2 μM ChlI, 0.4 μM ChlH in 50 mM Mops/KOH, 0.3 M glycerol, 1 mM DTT, 10 mM free Mg^2+^, *I*=0.1 with KCl and 8 μM D_IX_, pH 7.7, at 45°C. The curves can be described by eqn (2). *n*_d_ was held at 0.4 (i.e. one binding site on ChlH for Gun4) based on the fit in (**A**) with characterizing parameters WT ChlH *K*_app_=0.39±0.14 μM and Δ159ChlH *K*_app_=0.05±0.03 μM.

To characterize the interaction between ΔN159ChlH and Gun4, a FRET experiment was performed. Here, ChlH was labelled with Atto 488 NHS ester, and Gun4 with Alexa Fluor 532 maleimide. Figure 5(A) shows the quenching of the emission of Atto 488-labelled ChlH emission at 524 nm. Both WT and ΔN159ChlH show very similar FRET quenching behaviour, and when analysed using eqn (1), the *K_d_* of the interaction with Gun4 is essentially identical. Thus, deletion of the N-terminal head region of ChlH does not affect the binding of Gun4 and the binding site for Gun4 must reside in the remainder of ChlH.

To assess the ability of the ΔN159ChlH subunit to form part of a MgCH catalytic complex and to interact with the Gun4 enhancer protein, steady-state assays were performed in the presence of ChlI, ChlD and Gun4. WT ChlH and ΔN159ChlH from *T. elongatus* were assayed for *in vitro* MgCH activity by continuously monitoring fluorescence from the MgD_IX_ product at 580 nm. The assays were performed using recombinant H, I and D subunits from *T. elongatus*, at 45°C [15]. Figure 5(B) shows the steady-state rates of MgCH activity for WT ChlH (●) and ΔN159ChlH (○) in the presence and absence of Gun4. As expected from previously published work [21], the steady-state rate of WT MgCH increases up to 1.6-fold with increasing concentration of Gun4. ΔN159ChlH protein has 10% activity compared with WT, which in presence of saturating concentrations of Gun4 is increased to ~24% of WT activity. The *K*_app_ for the Gun4–ΔN159ChlH MgCH complex is ~8-fold lower compared with WT, indicating that saturation of the MgCH complex occurs at a lower concentration of Gun4. Removal of the N-terminal head region of ChlH appears to enhance Gun4 binding to the core chelatase complex, possibly by making the binding site more available.

## DISCUSSION

The H subunit binds the Proto substrate and the MgProto product of the reaction catalysed by MgCH, which requires the formation of a transient H–I–D complex and the hydrolysis of 14 MgATP^2−^ molecules [12]. Thus, interactions with the I and/or D subunits are part of ChlH function, as is the formation of a complex with the Gun4 protein [19,20], and with the next enzyme in the pathway, MgProto methyltransferase [27–30]. Nonetheless, it is not obvious why ChlH is such a large protein, comprising 1326 amino acids and 148 kDa for the *T. elongatus* subunit, for example. It was proposed in [18] that the hollow ‘caged’ structure of ChlH encloses the MgProto product, protecting it from photo-oxidation and channelling it to the methyltransferase. Thus, the need for a protective enclosed structure could account for the extra size of ChlH relative to its BchH counterpart from anaerobic photosynthetic bacteria where harmful light/oxygen combinations are less likely. However, even the BchH subunits from the purple photosynthetic bacteria such as *Rba. capsulatus* consist of 1189 residues and 129 kDa, so this large three-lobed structure, determined by single particle reconstruction at a resolution of 25 Å [17], is required for its core catalytic functions. These require interactions with porphyrins and the I and/or D subunits, but not, for example, with Gun4, since no homologue of this enhancer protein has been found in purple phototrophs.

The single particle/SAXS study of the *T. elongatus* ChlH protein [18] revealed that the majority of the protein forms a hollow globular structure, which is attached to an N-terminal ‘head’ domain through a narrow linker. The present work also showed that porphyrin binding and catalytic activity can be separated to some extent; N-terminal deletion of 565 residues (42% of the sequence) from the *Synechocystis* ChlH abolished enzymatic function, leaving porphyrin binding unaffected. Thus, normal catalytic function requires the N-terminal 565 residues, and the purpose of the present study was to narrow down the search for a catalytically essential region of ChlH by removing 159 residues from the N-terminus. Ideally, removal of this domain would leave the rest of ChlH unaffected structurally, if not functionally, and indeed the low-resolution structure of the ΔN159ChlH mutant and comparison with WT ChlH show that the N-terminal domain has been removed cleanly from the rest of the protein, leaving the rest of the structure apparently intact. This observation provides confirmation that that the head domain is indeed encoded by the ~17.6 kDa N-terminal fragment. Given that ΔN159ChlH appears to maintain its hollow globular structure with or without bound porphyrin and the protein appears to be correctly folded, ChlH appears to consist of two autonomous domains, joined by a narrow hinge region. We speculated that this Gly^127^–Phe^156^ region controls the binding of ChlH to other subunits or to the membrane, or it is involved in mobility of the head domain, possibly opening or closing the cavity within ChlH [18].

Analysis of dissociation constants shows that ΔN159ChlH is able to bind Proto and MgProto with normal affinities; the FRET data in Figure 5(A) indicate that the N-terminal truncation has not impaired the binding of Gun4 to this subunit. Chelatase assays with the H, I and D subunits show that the loss of the N-terminal domain has severely decreased MgCH activity, so this delineates the porphyrin binding and catalytically important parts of ChlH more precisely than achieved previously. The addition of Gun4 partly revives the MgCH activity of ΔN159ChlH, apparently compensating for the loss of the head domain to a limited extent. The ability of Gun4 to restore some activity to inactive ChlH has been noted before, when the *gun5-1* and *cch* mutations corresponding to A990V and P642L respectively in the *Arabidopsis* homologue [31] were introduced into the *Synechocystis* ChlH subunit, as A942V and P595L. Each of these mutations inactivates ChlH in MgCH assays, but the addition of Gun4 restored activity to 30–50% of WT (i.e. ChlH+Gun4) levels [32]. Gun4 does form a complex with ChlH [19,20], but this cannot be a result of interacting solely with the N-terminal domain, since it is absent from the ΔN159ChlH mutant. Nevertheless, it is possible that Gun4 binds adjacent to the N-terminal region perhaps promoting interaction of the I and D subunits with ChlH. Structural analysis of H–Gun4 complexes will help to clarify the functional role of Gun4 and its interaction with the ChlH subunit.

## Online data

Supplementary data
