# Supplementary material for: Structural and functional consequences of removing the N-terminal domain from the magnesium chelatase ChlH subunit of Thermosynechococcus elongatus
Source: Biochem J. 2014 Dec 5;464(Pt 3):315–22. doi: 10.1042/BJ20140463 (PMC4255732; doi:10.1042/BJ20140463)
Supplement: Supplementary data [file bj4640315ntsadd.pdf]

SUPLEMENTARY FIGURES

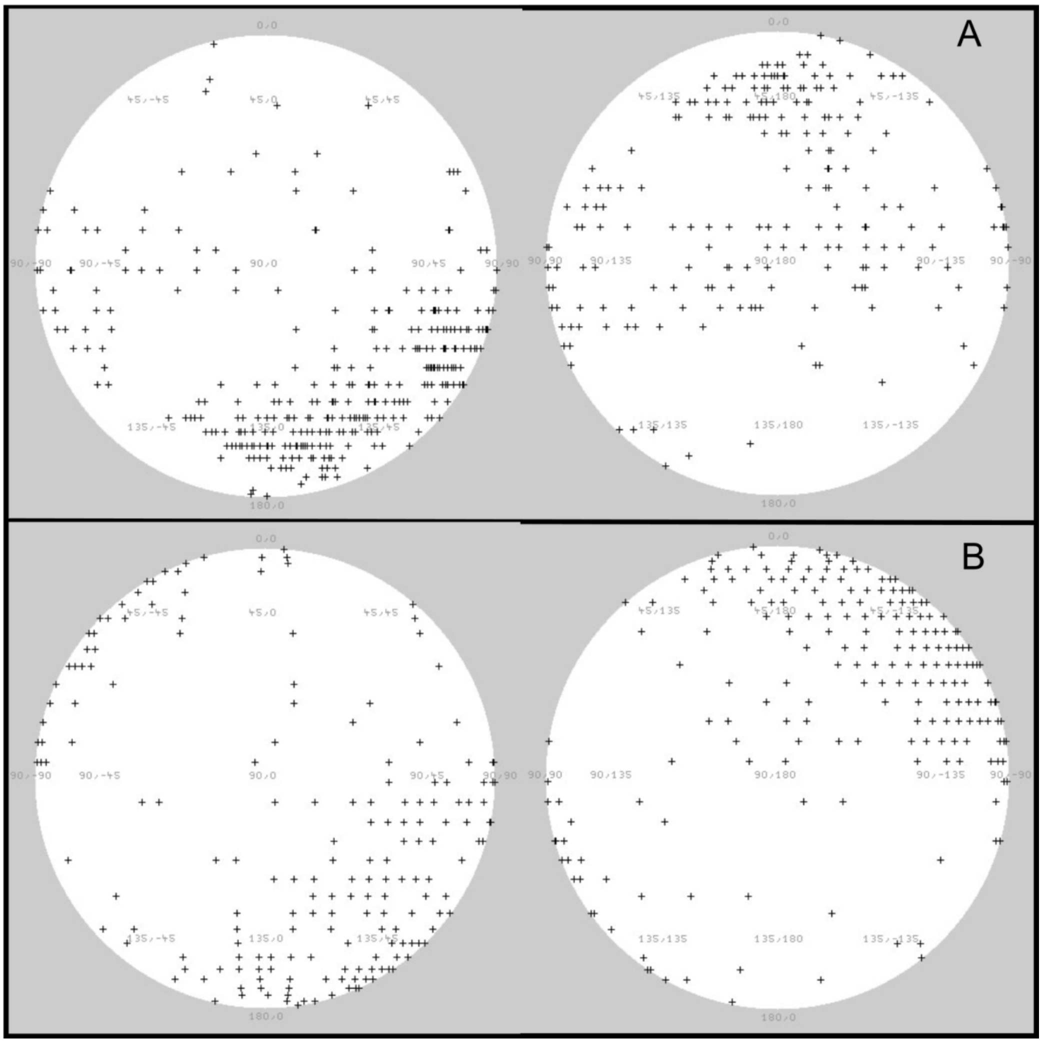

Figure S1. Euler angle distribution of the image classes used to reconstruct the final 3D models of the *T. elongatus* ΔN159ChlH protein (A) without and (B) with bound porphyrin.

The Euler angle definition in IMAGIC 5 was used for displaying the distribution of averaged classes. The positions of individual classes are indicated with a cross on the surface of a sphere, on which Euler angles β,γ are labelled. The left panel is a front view and the right panel is a back view of the sphere.

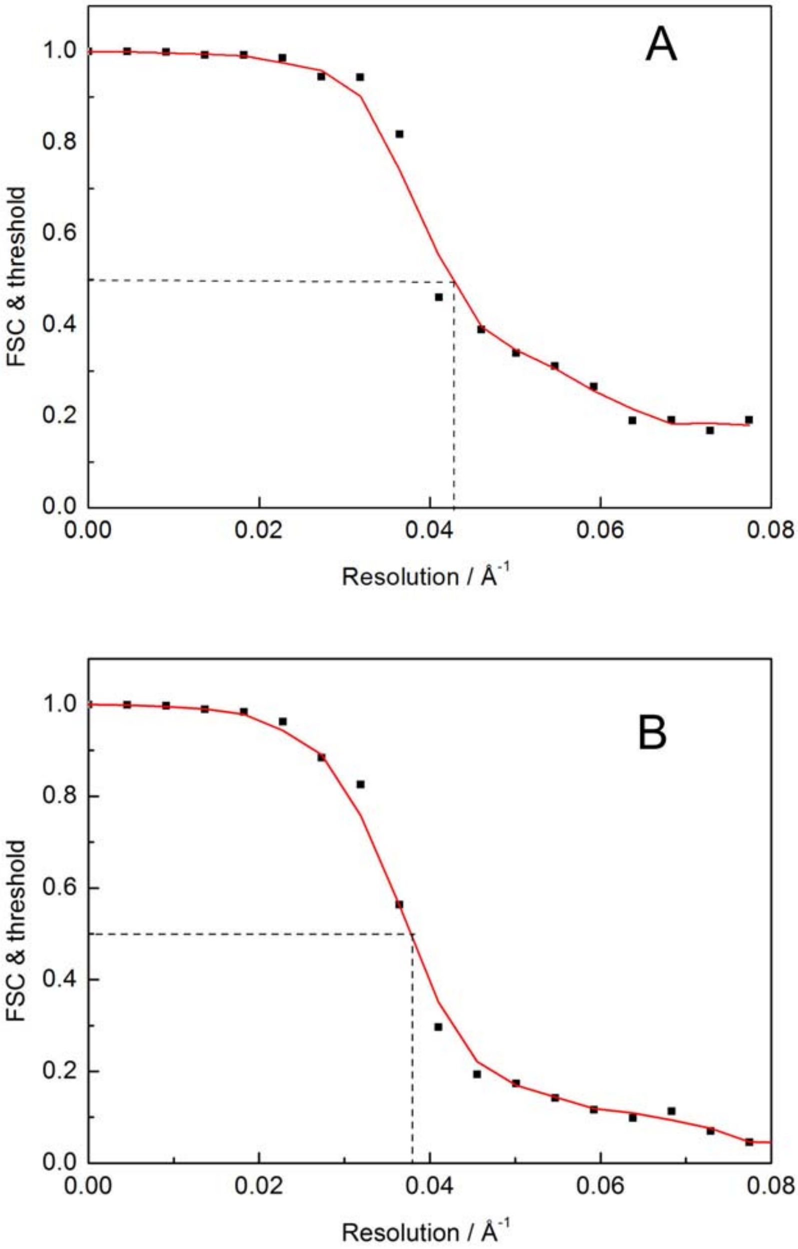

Figure S2. Determination of resolution at 0.5 Fourier shell correlation for the *T. elongatus* ΔN159ChlH protein (A) with (27 Å) and (B) without (23 Å) bound porphyrin.

.

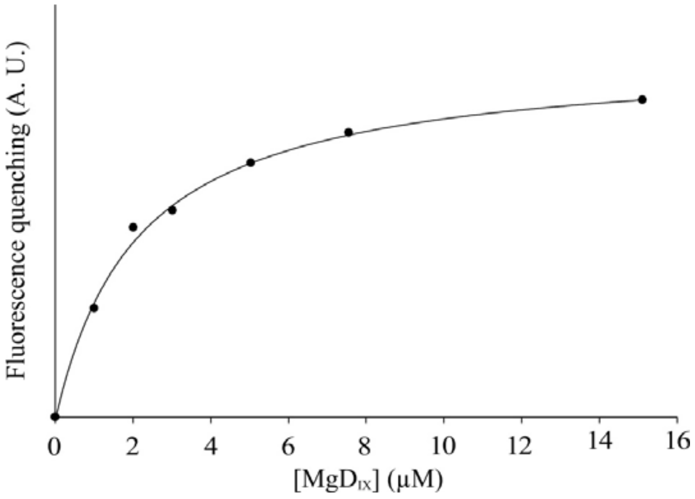

Figure S3. Product binding curves for wild-type *T. elongatus* ChlH

Quenching of *T. elongatus* ChlH protein fluorescence by Mg-deuteroporphyrin IX. 0.1 μM of purified ChlH was incubated with 0 – 15 μM MgD_{IX} and incubated at 34°C. Using the excitation wavelength of 295 nm fluorescence emission scans were recorded at 34°C. The curve shows single-site binding.
